# Can people apply ‘FAST’ when it really matters? A qualitative study guided by the common sense self-regulation model

**DOI:** 10.1186/s12889-019-7032-6

**Published:** 2019-05-28

**Authors:** Alison Morrow, Christopher B. Miller, Stephan U. Dombrowski

**Affiliations:** 1 Division of Psychology, School of Natural Sciences, University of Stirling, Stirling, FK9 4LA UK; 2 Faculty of Kinesiology, University of New Brunswick, Fredericton, NB Canada

**Keywords:** Stroke, Act FAST campaign, Common sense self-regulation model, Qualitative analysis

## Abstract

**Background:**

Early identification of stroke symptoms and rapid access to the emergency services increases an individual’s chance of receiving thrombolytic therapy and reduces the likelihood of infirmity. The UK’s national stroke campaign ‘Act FAST’ was developed to increase public awareness of stroke symptoms and highlighted the importance of rapid response by contacting emergency services. No study to date has assessed if and how people who experienced or witnessed stroke in line with the campaigns’ symptoms of the FAST acronym (i.e., facial weakness, arm weakness, slurred speech, and time) may use this FAST in their response.

**Methods:**

Semi-structured interviews with 13 stroke patients and witnesses were conducted. Interviews were theory-guided based on the Common Sense Self-Regulation Model, to understand the appraisal process of the onset of stroke symptoms and how this impacted on participants’ ability to apply their knowledge of the FAST campaign.

**Results:**

The majority of patients (*n* = 8/13) failed to correctly identify stroke and reported no impact of the campaign on their stroke recognition and response. Inability to identify stroke, perceiving symptoms to lack severity and lack of control contributed to a delay in seeking medical attention.

**Conclusion:**

Stroke witnesses and patients predominantly fail to identify stroke which suggest a lack of FAST application when it matters. Inaccurate risk perceptions and lack of physical control both play central roles in influencing the formation of illness representation not associated with an appropriate emergency response.

## Background

Identifying stroke symptoms early and receiving rapid medical attention is imperative to reduce the risk of neurological ailment, long-term cognitive and motor infirmity. Despite this, delays in contacting the emergency medical services (EMS) following stroke have been found to range from between 3 to 6 h [1] which greatly impairs the likelihood of receiving effective treatment such as thrombolytic therapy. When eligible patients receive thrombolytic therapy within 4.5 h of the onset of symptoms it can have a beneficial impact on patient outcomes [2–4]. Although an estimated 15–20% of stroke patients admitted to hospital are eligible for thrombolytic therapy [5], only 3.8% of patients received it in the UK [6]. One factor that prevents stroke patients from receiving the best possible care for stroke is pre-hospital delay [7].

Pre-hospital delay may be explained by lack of knowledge of stroke symptoms as well as the appropriate response action to take [8]. A systematic review found that the general public showed a lack of knowledge of the two most common stroke symptoms [9]. Additionally, although the majority of members of the public indicated that they perceive a stroke to be a serious incident that requires an ambulance, initial contact is often made with a general practitioner and contributes to pre-hospital delays [10]. In light of this, the Department of Health (2009) introduced the “Act FAST” campaign to target the general population to increase stroke symptom awareness and highlight the importance of rapid response by calling emergency medical services. The “FAST” acronym was developed based on the three most common and stroke specific symptoms (facial weakness, arm weakness and slurred speech) as well as the importance of responding to these symptoms immediately (time). Despite mass coverage of the campaign for several years strong evidence of campaign effectiveness is mixed [9, 11–13].

A previous qualitative study that explored the perceived effectiveness and impact of the Act FAST campaign focused on stroke patients, stroke witnesses and clinicians [14]. Despite clinicians reporting that the campaign improved stroke awareness, they assumed that this awareness had little, or no, effect on patients’ and witness’ response behaviour. One of the limitations of this study was that patients experienced a variety of symptoms, which were often not in line with those depicted by campaign. Although there are many possible explanations for such delays and the campaigns apparent lack of effectiveness, it is still unknown what determines how and why people seek medical help following the onset of stroke symptoms. To fully understand the campaigns aptitude to reducing delay, it is important to initially understand how individuals experience and respond to health threats.

It has been assumed that the decision process of whether to access health care services and respond to symptoms depend on how an individual perceives the illness [15]. Emerging somatic changes can alert an individual to the onset of a health threat or an illness which leads them to self-diagnose and make sense of the situation based on what they think is happening to them [16]. The Common Sense Self-Regulation Model (CS-SRM) [17], describes this process and assumes that it is the way that an individual perceives a somatic change that influences their appraisal, and consequently, how they respond. The CS-SRM proposes that a variety of factors contribute to an individual’s mental representation of their illness which influences their understanding of the threat, known as an illness representation. The illness representation involves five perceptions of the illness: what the illness is (identity or label), how long it is going to last (timeline), the believed consequences (consequence), its cause, and whether the threat can be controlled/cured. Additionally, an individual’s illness coherence [18], for example whether a person thinks about the threat in a logical way, also plays a significant role.

The CS-SRM [17] has previously been applied to guide research on health care seeking behaviour [19] and more specifically, to gain an insight into the appraisal process of stroke symptoms and how this might influence response behaviour. Dombrowski et al., (2012) used the CS-SRM as a theoretical tool to analyse qualitative data and explore how illness representations can influence witnesses’ responses to the onset of stroke symptoms [20]. This study found that failure to identify stroke often led participants to form illness representations that were not associated with an emergency and ultimately cause delay in contacting the emergency services.

Building on previous research, this study applied the CS-SRM during data collection and analysis to prompt facets of the stroke appraisal process. This study focuses on stroke patients and witnesses who were confronted with symptoms in line with the FAST campaign and therefore had reason to apply campaign knowledge to guide their response behaviour.

### Aim

To explore whether and how stroke witnesses and patients faced with stroke symptoms in line with the FAST acronym (facial weakness, arm weakness and speech disturbance) apply the knowledge of the FAST campaign and examine the stroke appraisal process.

## Methods

### Participants & study design

Semi-structured interviews with adults (18 years or older) who experienced or witnessed symptoms in line with the FAST campaign (facial weakness, arm weakness and speech disturbance) in the past year were conducted. Stroke patients and witnesses were recruited between April 2015 and June 2015 with the help of the third sector charity Chest Heart & Stroke Scotland. Eligible participants were initially identified (using charity records) and approached by Stroke Nurses within the charity, either during home visits or via telephone. Eligible participants were given background information of the study, research aims and reasons for doing the research to inform their decision to participate. Contact details of those who were interested were passed on to the research team and contacted to arrange an interview. Nurses only recruited participants who had no severe cognitive impairment or communication difficulties to ensure that their ability to remember the experience would not be hindered and that there was no misinterpretation during the interview process.

### Procedure

One-off semi-structured interviews using a topic guide were conducted. Prior to participation, all participants provided written and informed consent. Interviews were conducted at the home of the participant. Field notes were made during each interview, as well as being audio-recorded, and subsequently transcribed and anonymized. Transcripts were not returned to participants. This study gained ethical approval from the University of Stirling Psychology Ethics Committee. The CS-SRM [17] was used to guide data collection to prompt facets of the processes involved in making sense of a health threat. The topic guide consisted on two sections. The first section included questions around different facets of the illness representation, including:

- Identity/Cause: At the time, what did you think was happening to you? / Did you have any understanding of what may have caused the symptoms?
- Coherence: Did the situation make sense to you?
- Consequence: Did you think about what the consequences of the symptoms might be?
- Timeline: How long did you think the symptoms would last?
- Control: Did you feel like there was something that you could do to help with the symptoms?

The second section included questions around the Act FAST campaign, including:

- Were you aware of, or did you have any knowledge of, the FAST campaign before the stroke?
- Do you think this campaign had any impact on your decision/response?

### Data analysis

Transcripts were analysed applying theory-guided content analysis [21] led by the CS-SRM [17] theoretical framework in line with previous research [20]. This methodology was chosen as it aligns with the underlying theory around which this study was designed and allows exploration of theoretical facets prompted as well as the generation of additional explanations outside of the framework where needed. Transcripts were analysed and responses were allocated to facets of the theoretical framework (i.e., identity/cause, coherence, consequences, timeline, and control). Quotes from participants are abbreviated throughout indicating whether they were a patient (P) or a witness (W), participant number, speed of response time (i.e., the time taken between the onset of symptoms and initiating a response) and response behaviour (i.e., contacting the EMS [telephone 999] or primary care surgery [General Practitioner: GP]).

## Results

Eight stroke patients (4 female, 4 male) and five stroke witnesses (4 female, 1 male) were recruited. Seven spouses and family members (3 male, 4 female) were present during interviews. Participants were aged between 43 and 82 years (*Mean* = 69 years). All stroke experiences occurred at homes and the relationships of the witnesses were either wife (*n* = 4) or husband (*n* = 1). Interviews lasted between 21 and 58 min (*M* = 27 min). The strokes occurred between 2 to 51 weeks prior to interview dates (*M* = 15 weeks). Participant response times varied significantly, ranging from 5 min to 5 h (*M* = 1 h 15 min). Stroke patient health service contacts were all made to the EMS (*n* = 8). Seven contacts were made by other individuals on behalf of the patient and one patient contacted the EMS themselves. Three stroke witnesses contacted the EMS and the remainder (*n* = 2) contacted the GP.

### Identity/cause

Eight participants (*n* = 8/13) were aware of the FAST campaign prior to observing or experiencing the stroke and two participants perceived that the campaign impacted their response action.

*“I feel I would have done it anyway whether I had seen it or not” (WP1, 5 minutes, 999).*

*“Cos’ obviously my face, arm, speech, I had everything! It definitely done it for me!” (PP5, 5 minutes, 999)*

Five (*n* = 5/13) participants reported successfully identifying the observed symptoms as stroke.

*“I was quite persuaded it was a stroke” (PP11, 10 minutes, 999).*

These participants almost immediately responded to the symptoms by calling the EMS. One participant reported that their ability to correctly identify a stroke and take the appropriate action by calling 999 came from the knowledge that they had gained from the FAST campaign.

*“I had seen the adverts on the tele[...] they go through what happens and I saw her face and she was just talking gibberish. I thought of the adverts on the tele so that’s how I knew” (WP10, 5 minutes, 999).*

However, two of the youngest participants in their 40s, felt that they failed to correctly identify that they were having a stroke because they did not associate themselves with the campaigns, or their own, representation of a stroke.

*“I didn’t really associate myself with that person on the TV…it was furthest from my experience [… ] maybe in the advert you can have a younger guy in it as well…it doesn’t just happen to old people and people need to know that” (PP8, 50 minutes, 999).*

*“Something in my head did say I’m having a stroke. But the other side said, I canny be having a stroke, I’m only 48!” (PP6, 45 minutes, 999).*

The remaining participants (*n* = 3/5) misinterpreted the symptoms they observed and tended to associate them to other non-urgent health conditions. This caused the witnesses to dismiss the symptoms as a threat that had already received medical attention and therefore assumed that they were under control.

*“I thought it was something to do with his heart because he has already had a heart attack” (WP4, 4 hours, GP).*

Additionally, most stroke patients reported no understanding of what was happening to them. As a result they were unable to identify the symptoms or the appropriate response action to take by applying the FAST test.

*“I had no idea that my face was distorted or that my speech was slurred. It was my husband that pointed this out to me […] I just thought, I’ve fell out of bed how on earth did I do that” (P2, 10 minutes, 999).*

It was also common for participants to lack association between the symptoms they observed and the symptoms presented in the campaign. This mismatch between their experience and those advertised led witnesses to eliminate stroke as a potential cause of the symptoms.

*“His symptoms weren’t as…severe as they show on the tele” (WP7, 5 hours, 999).*

### Coherence

A number of participants made statements that suggested that they did not have a clear understanding of the symptoms they were observing.

*“I knew she was having a stroke but I didn’t really know why. When I had left her to go to bed she was fine, she didn’t say she wasn’t feeling well…So I didn’t really understand” (WP10, 5 minutes, 999).*

*“One doesn’t just collapse on the floor for no reason […] I really didn’t expect this” (WP13, 10 minutes, 999).*

One participant (*n* = 1/8) appeared to think about their symptoms in a logical way, leading them to correctly identify what they were experiencing and then base their response action on the knowledge that they had of stroke.

*“It made horrific sense to me. It said, “Here’s the onset of a stroke” […] I knew the symptoms were serious and I needed to call [999]” (PP11, 10 minutes, 999).*

### Consequences

An expectation of what would follow, for example whether participants perceived that the consequences of the symptoms would be minor or major, played an important role in the identification of the need to call the EMS.

*“[I thought about] the dire effects of a stroke, you know. The helplessness, it just flew through my mind” (WP1, 5 minutes, 999)*

There were circumstances where participants were not fully aware of what was happening, but reacted to their natural instinct that the situation was ominous.

*“I just knew it was a hearty type thing” (WP13, 10 minutes, 999).*

Conversely, a number of participants perceived the consequences of the symptoms to lack severity and assumed that they did not warrant medical attention. This tended to reflect a delay in seeking medical attention.

*“I was putting it down to him just being a bit tired and clumsy” (WP4, 4 hours, GP).*

*“I felt my arm drop, just like they show you on the tele but I thought I had just dropped something” (PP9, 1 hour, 999).*

### Timeline

Ten (*n* = 10/13) participants, identified that they were aware their symptoms were long-term, even if they did not identify them with a stroke.

*“I just knew it wasn’t [going to take] a couple of pain killers and you’ll be fine” (PP2, 10 minutes, 999).*

The expectation when you take a pain killer is that the pain or discomfort that you are feeling will go away in a short period of time. This highlights that the participant believed that their symptoms would not pass which seemed to influence immediate response.

Participants with the lengthiest response times assumed that the symptoms were acute and would not endure.

*“I thought you know, after he had a nap he would be feeling a lot better” (WP7, 5 hours, 999).*

*“At the time I thought they [facial weakness and arm weakness] were just going to go away” (WP4, 4 hours, 999).*

*“I just thought this is going to pass. It’ll pass, it’ll pass” (PP9, 1 hour, 999).*

### Control

The feeling of lack of control often prompted help-seeking behaviour in witnesses. All participants (*n* = 13/13) reported that they could not do anything to help or control the symptoms and therefore had to seek help from somewhere else, which in most instances was the EMS.

*“I thought I needed help. I need 999” (WP1, 5 minutes, 999).*

*“I knew I couldn’t help her” (WP10, 5 minutes, 999).*

*“I was concerned about getting seen to, and quickly […] because I am well aware that […] if you get it attended to quickly, there is a good chance that things can be rectified” (PP11, 10 minutes, 999).*

Six participants (*n* = 6/13) reported that they were aware they required medical attention however they were physically unable to respond.

*“I couldn’t help myself. I thought I was finished” (PP8, 50 minutes, 999).*

In most cases (*n* = 5/6) this lack of control did not seem to result in a delay in presenting to the EMS because they received assistance from a witness. Delays in calling the EMS seemed to only be influenced by the inability to control the situation when the stroke patient had no witness to act on their behalf. The campaign is mostly useful for witnesses observing symptoms.

*“It’s the people that are witnessing the strokes that can do something about it. They are more in power. You have got to rely on them” (PP3, 4 hours, 999).*

## Discussion

This study highlights that both stroke patients and witnesses typically do not report applying their knowledge of the FAST campaign when faced with stroke symptoms that are in line with the FAST acronym. The main reasons for the lack of reported application of the FAST knowledge included a lack of association between perceived symptoms and stroke, not seeing the campaign as personally relevant, and lack of campaign awareness.

Overall, although 8/13 participants were aware of the FAST campaign prior to experiencing or witnessing stroke symptoms, only two believed that this impacted their decision to respond in any way. A contributing factor of this was that approximately half of all witnesses and stroke survivors were unable to correctly identify that they were observing, or experiencing, a stroke. In both stroke patients and witnesses, failure to correctly identify symptoms as those of a stroke often contributed to a delay in responding. Lack of association led to participants misidentifying what was happening and they were consequently unable to apply the knowledge of the FAST campaign.

For the FAST campaign to be more successful in reducing delay in presenting to the emergency services, individuals initially need to be able to relate to the campaign during their experience. The three main factors that appeared to hinder participant’s ability to relate to, and apply, the FAST campaign were symptom experience, knowledge of stroke symptoms, and age. Similarly to other research findings [22] participants regularly misunderstood aspects of the campaign. A recurring theme was that there was a mismatch between their experience and the campaigns representation of stroke. This may have resulted in participants dismissing stroke as a potential cause of the symptoms. Additionally, participants were often unaware of the symptoms of stroke and therefore did not feel that the information the campaign communicated was relevant to them. Even when participants had almost all symptoms of stroke [23], they frequently reported that they were not aware that they were at an increased risk. This resulted in participants regularly assigning symptoms to a previously diagnosed condition and forming an illness representation associated with something minor that did not require immediate medical attention. Finally, the two youngest participants believed that they had reasonable knowledge of the campaign and the message it portrayed, however they did not apply this knowledge during their experiences. It appeared that the reason that these participants did not associate themselves with the campaign was because the demographics of the individuals used in the campaign did not reflect their own situation. The campaign generally portrays older individuals experiencing stroke which could result in younger individuals not being able to relate to the campaign and therefore not feel like they are at risk of stroke. This is a particular issue as the trend of stroke is changing, with increasing numbers of young people being affected by stroke every year. Currently, up to 25% of strokes in the UK occur in adults under the age of 65 [24] which highlights the importance of the message being portrayed that stroke can happen to anyone, not just older adults.

Lack of physical control, which was predominantly a concern for stroke patients rather than witnesses, also appeared to prevent participants from applying the FAST knowledge. One participant reported that although they were aware that they were having a stroke and reflected on their knowledge of the campaign, they were physically unable to follow through with effective response behaviour. This suggests campaigns that impact may be limited to witnesses of stroke symptoms rather than those who are experiencing stroke. This is reflected in calls made to EMS with stroke patients responsible for 2–7% compared to 93–98% of witness calls [25, 26].

The current study suggests several avenues for improving the FAST campaign. A closer match between the manner in which stroke symptoms are perceived and the display of these symptoms in the campaign would add value. The CS-SRM [17] used in this study was found to be a useful model to explore symptom perceptions and cognitions and could serve as a theoretical background for revising this and other awareness raising campaign. Moreover, at present, the campaign generally portrays older adults. Adapting the campaign to include younger adults in everyday situations, such as at work or socialising with friends may help raise awareness of stroke.

### Strengths & limitations

This study has benefits over other research in the field examining the effects of the FAST campaign [14, 20] as it includes both stroke patients and witnesses, and focuses on those who experienced or witnessed symptoms in line with the FAST campaign. Understanding the experiences of stroke patients and witnesses is crucial to give a more accurate account of the campaigns impact, as they constitute the group who could have benefited from the content of the FAST campaign. The use of psychological theory and the CS-SRM as a theoretical tool [20] to understand participants’ appraisal of their stroke experience is a particular strength, as research of this kind can be used to guide theory-based interventions. One of the main limitations of this study was that the data is reliant on participants self-report and interpretations of events, some of which happened up to a year previous to the interviews. This creates potential for recall bias due to potential mild cognitive impairment as well as a prolonged period of time between the event and the interview. Results of qualitative research designs are not generalisable to the overall population of patients who experience or those who witness stroke. Additionally, due to the inclusion and exclusion criteria, the population of stroke patients did not include those who were affected by severe stroke. Results serve as a starting point to understand the impact of the FAST campaign on the behaviour of stroke patients and witnesses and further samples may be required.

## Conclusions

Participants were frequently unable to apply the FAST message and knowledge during their own experience. This suggests that although the campaign is practical and valuable in theory, those who need it most often do not apply it in practice. This appeared to be largely guided by inaccurate risk perceptions and the inability to identify with stroke in general. The content of the campaign itself could be improved to increase its effectiveness.

## Data Availability

Excerpts of the transcripts relevant to the study can be requested from the corresponding author.

## Abbreviations

CS-SRM: The Common Sense Self-Regulation Model
EMS: Emergency medical services
FAST: Facial weakness, arm weakness, Slurred speech and Time
GP: General practitioner
P: Patient
W: Witness
